# Severe community-acquired pneumonia in general medical wards: outcomes and impact of initial antibiotic selection

**DOI:** 10.1186/s12890-019-0944-1

**Published:** 2019-10-16

**Authors:** Phunsup Wongsurakiat, Napat Chitwarakorn

**Affiliations:** 1 Division of Respiratory Disease, Department of Medicine, Siriraj Hospital, Mahidol University, Bangkoknoi, Bangkok, 10700 Thailand; 2 Bamrasnaradura Infectious Disease Institute, Tiwanon Road, Amphur Mueng, Nonthaburi, 11000 Thailand

**Keywords:** Antibiotic, Hospital-free days, Intensive care unit, Mortality, Outcome, Treatment failure

## Abstract

**Background:**

Most international guidelines recommend empirical therapy for community-acquired pneumonia (CAP) to be based on site of care. Some patients with severe CAP are managed in general wards because of limited intensive care unit (ICU) bed or because of unrecognition of the pneumonia severity. Appropriate initial antibiotic treatment for severe CAP outside ICU has not yet been established. This study aimed to determine the prevalence and the impact of initial antibiotic selection on the outcomes of patients with severe CAP who were admitted and managing in general wards.

**Methods:**

This prospective observational study included consecutive patients hospitalized for presumed CAP in general wards over a 1-year period. Severe CAP was identified using the 2007 Infectious Diseases Society of America (IDSA)/American Thoracic Society (ATS) criteria. Initial antibiotic treatment in the first 24 h were collected. The primary outcome was the rate of unfavorable outcome (composite outcome of treatment failure and in-hospital death). The secondary outcome was the number of hospital-free days assessed 30 days after enrollment into the study.

**Results:**

There were 94 patients hospitalized with CAP of which 50 (53.2%) patients were compatible with severe CAP. An etiologic diagnosis was found in 43 (45.8%) patients. The most common pathogens identified in patients with severe CAP were *Staphylococcus aureus* (28.6%) and *Klebsiella pneumoniae* (28.6%), followed by *Pseudomonas aeruginosa* (17.9%). Patients with severe CAP had significantly more positive blood culture than patients with non-severe CAP (24% VS 4.5%; *p* = .008). Initial antibiotic treatment were discordant with the IDSA/ATS guidelines in 42% of all patients hospitalized with CAP, and 52% of patients with severe CAP. Multivariate analysis revealed that age (OR 1.1, 95% CI 1.01–1.1) and initial antibiotic treatment discordant to guidelines for severe CAP in ICU (OR 4.6, 95% CI 1.3–17.1) were independent risk factors of the unfavorable outcome of patients with severe CAP. Patients with unfavorable outcome had lower number of hospital-free days than patients with favorable outcome (5.2 ± 8 days VS 18 ± 7.1 days; *p* < .001).

**Conclusions:**

Patients with severe CAP outside ICU should be recognized for appropriate initial antibiotic selection to improve outcomes.

## Background

Community-acquired pneumonia (CAP) is a leading cause of hospitalization and death worldwide [1–5]. Severe CAP is a group of patients who have severe disease with poor outcomes and requiring a higher level of care [6, 7]. Several criteria have been proposed to define severe CAP. Most reports have defined it simply as CAP that necessitates intensive care unit (ICU) admission.

Initial treatment of CAP is usually empirical, because the microbial etiology cannot be predicted on the basis of clinical presentation. Most international guidelines recommend empirical therapy that is based on the location of care, with specific recommendations for those managed as outpatients, as inpatients, and in ICUs [7–10]. Adherence to initial antibiotic selection guidelines was reported to be associated with improved survival and reduced duration of mechanical ventilation of patients with severe CAP in ICU [11–14].

ICU admission policies and the availability of ICU beds varies widely across the world. The shortage of ICU beds is a daily problem in many hospitals, especially in under-resourced countries [15–18]. Moreover, there is a category of severe CAP classified by the 2007 Infectious Diseases Society of America (IDSA)/American Thoracic Society (ATS) minor criteria [7] that may be under-recognized because patients may initially present with subtle findings [7, 19]. As many as 45% of patients with CAP who ultimately required ICU admission were initially admitted to a non-ICU setting [20]. As a result, in many hospitals, a substantial number of patients with severe CAP might be managed mainly in general wards due to either limited number of ICU beds or under-recognition of pneumonia severity.

To the best of our knowledge, no study has yet specifically focused on the outcomes and impact of initial antibiotic treatment in patients with severe CAP that are admitted to general wards. One previous study reported that using IDSA/ATS minor criteria to early identify patients at risk for severe CAP combined with an aggressive emergency department resuscitation bundle, including initial antibiotic treatment protocol, may reduce mortality in this group of patients [21]. Accordingly, we set forth to study the prevalence and outcomes of patients with severe CAP who were admitted to and managed mainly in general wards, and to evaluate the impact of initial antibiotic selection on outcomes. Our working hypothesis was that the discordant site of care (i.e., general medical wards) would influence initial antibiotic selections that are discordant with IDSA/ATS initial antibiotic selection guidelines for patients with severe CAP in ICU, and that this might contribute to poorer outcomes.

## Methods

### Study population

This study is a secondary analysis of the data collected from a prospective observational study conducted from November 2012 to November 2013 at a 2500-bed university-based hospital in Thailand. The study aimed to determine the prevalence and outcomes of healthcare-associated pneumonia in the authors’ hospital. Our hospital has two sections: a private section and a general section. In the general section, we have around 2200 inpatient beds. The Department of Medicine has 462 inpatient beds comprise 160 general medical ward beds, 186 private beds, 76 special disease beds (such as stroke unit, bone marrow transplant unit) and 40 critical care beds (15 general ICU beds, 10 critical respiratory care beds and 15 cardiac care beds). This study was performed in 8 general medical wards affiliated with the Department of Medicine (160 beds).

Each general medical ward had a 20-patient capacity. Patients were managed by a medical team consisted of an attending physician, 1 third year resident in general internal medicine, 2 first year residents in general internal medicine and 3 to 4 interns rotated to work in the Department of Medicine. Attending physicians were board certified in internal medicine, with minimal critical care training. The medical team were rotated monthly. There were approximately 4 nurses and 4 nurse assistants doing nursing care for the patients.

Due to a shortage of ICU bed, patients who required mechanical ventilation were often admitted directly to a general medical ward. Patients mechanically ventilated on the wards were managed by the same attending physicians that treated non-ventilated patients on the wards. Upon request, some patients were continuous monitoring with electrocardiography, noninvasive blood pressure monitoring and oxygen saturation by pulse oximetry.

Consecutive patients admitted to 1 of 8 general medical wards affiliated with the Department of Medicine, were potentially eligible for this study. Patients that met all of the following criteria were included: age > 18 years, admitted from outside the hospital for presumed pneumonia with symptoms of acute respiratory infection (fever, cough, pleuritic chest pain, or dyspnea), and presence of new infiltrate on chest radiograph. Patients that were pregnant, that were enrolled in another trial, that had received immunosuppressants or long-term corticosteroid therapy, that had concomitant acquired immunodeficiency syndrome, that had undergone tracheotomy, or that had a preexisting medical condition with a life expectancy of less than 3 months were excluded. Enrolled patients were excluded during the follow-up period if patients were subsequently transferred to the ICU within 5 days after admission or the etiology of pneumonia was found to be mycobacteria or fungi.

### Study design and data collection

A protocol for data collection in the first 24 h was applied in all cases. Information that was collected included age, gender, smoking status, residence, comorbidities (pulmonary, heart, liver, neurologic, renal, neoplasms, and diabetes mellitus), enteral tube feeding, receiving chronic hemodialysis or wound care, hospitalization within the last 90 days, and broad-spectrum antibiotic therapy within the previous 90 days. Functional status was assessed using Eastern Cooperative Oncology Group (ECOG) scale of performance status [22].

The following clinical data were recorded: mental alterations, temperature, heart rate, respiratory rate, and blood pressure. For severity of illness, we assessed the need for mechanical ventilation and/or vasopressors within 24 h of admission. Chest radiographic findings were also documented (number of affected lung lobes, presence of pleural effusion). Recorded laboratory data were complete blood count, chemical parameters, and arterial blood gas analysis.

Microbiological studies included blood cultures, and collection of a sputum sample, pleural fluid, or bronchoalveolar lavage for Gram stain and culture, when possible. Only specimens obtained within 72 h before or after admission were included. Etiologic diagnosis was considered definitive in the following situations: isolation of a respiratory pathogen in a usually sterile specimen (blood, pleural fluid) or bacterial growth in bronchoalveolar lavage fluid (≥10^4^ cfu/ml). Etiologic diagnosis was considered presumptive when a predominant microorganism was isolated from a sputum sample (> 25 polymorphonuclear leukocytes and < 10 squamous cells per low-power field).

Initial antibiotic treatment prescribed within the first 24 h, and whether the clinician later had to modify the initial antibiotic regimen was recorded. Antibiotic prescriptions were left to the discretion of the attending physician and were not protocolized. No interventions relative to the prescribing physicians were effectuated prior to or during the study.

The study protocol was approved by the Siriraj Hospital Ethics Committee on Human Research (No. SIRB 391/2555-EC1). Written informed consent for inclusion in the study was obtained from each patient or the patient’s next of kin.

### Definitions

**Severe CAP** was defined according to IDSA/ATS criteria [7] as follows: (1) having 1 or more of the major criteria (invasive mechanical ventilation or septic shock with a need for vasopressors); (2) having 3 or more of the minor criteria (respiratory rate ≥ 30 breaths/min, PaO_2_/FiO_2_ ≤ 250 mmHg, multilobar infiltrates, confusion/disorientation, blood urea nitrogen (BUN) ≥20 mg/dL, WBC count < 4000 cells/mm^3^, platelet count < 100,000 cells/mm^3^, core temperature < 36 °C, hypotension requiring aggressive fluid resuscitation).

Healthcare-associated pneumonia (HCAP) was defined as patients with pneumonia that fulfilled any one of the following criteria: (1) hospitalization for more than 48 h in the last 90 days; (2) residence in a long-term care facility; (3) home infusion therapy within 30 days; (4) chronic dialysis within 30 days; or, (5) home wound care during the 30 days preceding admission.

### Antimicrobial therapy

Adequate antibiotic therapy was defined as treatment with at least one agent to which all recovered isolates were susceptible in vitro.

### Guideline-concordant and guideline-discordant antibiotic therapy

Empirical therapy within the first 24 h of hospitalization was evaluated for concordance or discordance to the 2007 IDSA/ATS guidelines for CAP [7] Patients were considered to have received guideline-concordant antibiotic therapy if they received initial treatment as outlined in Table 1, regardless of any additional antibiotic received. Patients with severe CAP were considered to have received guideline-concordant antibiotic therapy if they received initial treatment according to guidelines for patients with CAP in ICU. Patients who received all other antibiotic regimens were considered to have received guideline-discordant antibiotic therapy.

**Table 1:** 2007 Infectious Diseases Society of America/American Thoracic Society Guideline Recommendations for empirical therapy for community-acquired pneumonia

Inpatients, non-ICU treatment	
A respiratory fluoroquinolone^a^	
A nonantipseudomonal β-lactam^b^ plus a macrolide^c^	
Inpatients, ICU treatment	
A nonantipseudomonal β-lactam^b^ plus either azithromycin or a respiratory fluoroquinolone^a^	
If *Pseudomonas* is a concern	
An antipneumococcal, antipseudomonal β -lactam^d^ plus either ciprofloxacin or levofloxacin	
or	
The above β-lactam plus an aminoglycoside^e^ and azithromycin	
or	
The above β-lactam plus an aminoglycoside^e^ and a respiratory fluoroquinolone^a^	

ICU: intensive care unit

^a^Levofloxacin, or moxifloxacin

^b^Cefotaxime, ceftriaxone, ampicillin/sulbactam, or ertapenem

^c^Azithromycin, clarithromycin, or erythromycin

^d^Piperacillin-tazobactam, cefepime, imipenem, or meropenem

^e^According to Thai guidelines for the management of adults with community-acquired pneumonia, adding an aminoglycoside is optional. An aminoglycoside may be added to the initial antibiotic regimens only if multi-drug resistant *Pseudomonas* infection is suspected

### Outcomes

**Primary outcome** was the rate of unfavorable outcome, which included treatment failure (early, late, or both early and late) [23] or death during hospital admission. Early treatment failure was defined as clinical deterioration within 72 h of treatment (including a need for invasive mechanical ventilation or development of shock that was not present within the first 24 h after admission, or death). Late treatment failure was defined as one of the following criteria: persistent respiratory rate ≥ 30 breaths/min (non-intubated patients), a need for invasive mechanical ventilation or development of shock not present at baseline, radiographic progression (increase in pulmonary infiltrates of ≥50% compared to baseline), or death between 72 h and 120 h after the initiation of treatment**.**

**Secondary outcome** was the number of hospital-free days at 30 days after enrollment into the study. Number of hospital-free days was defined as the number of days from admission to day 30 that the patient was not admitted to the hospital (calculated by subtracting the length of hospital stay from 30).

### Statistical analysis

All data analysis was performed using SPSS Statistics software version 20 (SPSS, Inc., Chicago, IL, USA). Descriptive analysis was performed. Discrete variables are expressed as number and percentage (%), and continuous variables as either mean ± standard deviation (SD) or median and interquartile range (IQR). Proportions were compared using chi-square test or Fisher’s exact test for categorical variables, and nonparametric Mann-Whitney U-test or unpaired t-test for continuous variables. Statistical significance was defined as *p* < 0.05, and all reported *p*-values were two-tailed.

Multivariate analysis was performed with the backward stepwise elimination logistic-regression analysis model. The dependent variable was the rate of unfavorable outcome, and the independent variables were evaluable variables collected at admission that were associated with unfavorable outcome including in the univariate analysis (*p* < 0.2). Variables remaining in the multivariate analysis model that showed a *p*-value < 0.05 were considered significant.

## Results

### Patients

A total of 108 patients hospitalized with CAP were evaluated for eligibility. Ten of these patients were immunosuppressed and 4 were found to have mycobacterial infection, so these 14 patients were excluded from further analysis. Of the remaining 94 patients, 50 (53.2%) met the diagnostic criteria for severe CAP. Thirty-six of 50 patients (72%) required mechanical ventilation, and 14 (28%) were classified as severe CAP by IDSA/ATS minor criteria. Clinical characteristics, comorbidities, functional status, and clinical outcomes classified according to pneumonia severity are shown in Table 2.

**Table 2:** Subject demographic, clinical characteristics, and clinical outcomes by severity of community-acquired pneumonia (CAP)

	Severe CAP (*n* = 50)	Non-severe CAP (*n* = 44)	*p*-value
Age, y	70.9 ± 14.8	68.2 ± 20.3	.46
Gender (Female)	18 (36)	19 (43.2)	.48
Mechanical ventilation	36 (72)	0	<.001*
Minor criteria ≥3^a^	14 (28)	0	<.001*
Comorbid conditions:			
None / Single / Multiple	9 (18) / 21 (42) / 20 (40)	17 (38.6) / 17 (38.6) / 10 (22.7)	.05*
Prior antibiotic therapy^b^	21 (42)	22 (50)	.44
ECOG scale ≥2^c^	32 (64)	22 (50)	.17
Enteral tube feeding	6 (12)	3 (6.8)	.49
HCAP^d^	18 (36)	12 (27.3)	.36
Pleural effusion	3 (6)	8 (18.2)	.07
Albumin, g/dL	2.8 ± 0.6	3.1 ± 0.5	.01*
Globulin, g/dL	3.7 ± 0.8	4.1 ± 0.6	.001*
Treatment failure	23 (46)	4 (9.1)	<.001*
Death	14 (28)	4 (9.1)	.02*
Unfavorable outcome^e^	25 (50)	6 (13.6)	<.001*
Length of stay in hospital, d^f^	15.5 ± 12.8	8.3 ± 4.6	.003*
Hospital-free day, d ^g^	11.6 ± 9.9	19.7 ± 7.7	<.001*

Data are presented as mean ± SD or n (%), unless otherwise stated. ^a^IDSA/ATS 2007 minor criteria^7^. ^b^Prior antibiotic therapy: systemic antibiotic use in the 90 days prior to this admission. ^c^ECOG scale: Eastern Cooperative Oncology Group scale of performance status. ^d^HCAP: Healthcare–associated pneumonia. ^e^Unfavorable outcome includes treatment failure or death during hospital admission. ^f^ Length of stay in hospital in patients who survived to hospital discharge. ^g^Number of days from admission to day 30 that the patient was not admitted to the hospital. *Statistically significant difference

### Microbial etiology

An etiologic diagnosis was found in 43 (45.8%) patients, of which 15 (16%) were definitive and 28 (29.8%) were presumptive. The most common pathogens identified in patients with severe CAP were *Staphylococcus aureus* (28.6%) and *Klebsiella pneumoniae* (28.6%), followed by *Pseudomonas aeruginosa* (17.9%). The common pathogens identified in patients with non-severe CAP were similar to those identified in patients with severe CAP. However, patients with severe CAP had significantly more positive blood culture than patients with non-severe CAP (24% vs. 4.5%; *p* = 0.008), as shown in Table 3.

**Table 3:** Microbiological etiology by severity of community-acquired pneumonia (CAP)

	Severe CAP (*n* = 50)	Non-severe CAP (*n* = 44)	All patients (*n* = 94)		
			Blood or Pleural fluid	Sputum	Total
Positive blood culture	12 (24)	2 (4.5)	14 (14.9)	–	14 (14.9)
Etiological diagnosis	28 (56)	15 (34.1)	15 (16)	28 (29.8)	43 (45.8)
*Streptococcus pneumoniae*	2 (7.1)	0	2 (13.3)	0	2 (4.6)
Other *Streptococcus* spp.	3 (10.7)	1(6.7)	4 (26.7)	0	4 (9.3)
*Staphylococcus aureus*	5 (17.9)	3 (20)	3 (20)	5 (17.9)	8 (18.6)
*Haemophilus influenzae*	0	2 (13.3)	0	2 (7.1)	2 (4.6)
*Moraxella catarrhalis*	2 (7.1)	0	0	2 (7.1)	2 (4.6)
Enterobacteriaceae:	6 (21.4)	4 (26.7)	3 (20)	7 (25)	10 (23.3)
*Klebsiella pneumoniae*	5 (17.9)	2 (13.3)	2 (13.3)	5 (17.9)	7 (16.3)
*Escherichia coli*	1 (3.6)	0	1 (6.7)	0	1 (2.3)
Others	0	2 (13.3)	0	2 (7.1)	2 (4.6)
*Pasteurella* spp.	1 (3.6)	0	1 (6.7)	0	1 (2.3)
Potentially drug-resistant bacteria:	13 (46.4)	8 (53.3)	2 (13.3)	19 (67.9)	21 (48.8)
*Pseudomonas aeruginosa*	5 (17.9)	3 (20)	0	8 (28.6)	8 (18.6)
ESBL-positive Enterobacteriaceae*	4 (14.3)	1 (6.7)	1 (6.7)	4 (14.3)	5 (11.6)
*Acinetobacter* spp.	2 (7.1)	4 (26.7)	1 (6.7)	5 (17.9)	6 (13.9)
*Stenotrophomonas maltophilia*	0	1 (6.7)	0	1 (3.6)	1 (2.3)
Methicillin resistant *Staphylococcus aureus*	3 (10.7)	1 (6.7)	0	4 (14.3)	4 (9.3)
Polymicrobials	4 (14.3)	5 (33.3)	0	9 (32.1)	9 (20.9)

Data are presented as n (%). *ESBL: extended spectrum beta-lactamase

### Antibiotics administered

Initial antibiotic regimens are shown in Table 4. Of all 94 patients with CAP, 40 (42.6%) received initial antibiotic regimens that were discordant with IDSA/ATS guidelines. The most common guideline-discordant antibiotic regimen prescribed was antipseudomonas β-lactams without quinolone or macrolide. The risk factors for guideline-discordant initial antibiotic selection were severe CAP, prior antibiotics therapy, enteral tube feeding, HCAP, and treatment with antipseudomonas β-lactams as shown in Table 5.

**Table 4:** Initial antibiotics treatment and clinical outcomes by severity of community-acquired pneumonia (CAP)

	All Patients (*n* = 94)	Severe CAP (*n* = 50)	Non-severe CAP (*n* = 44)	*p*-value
Etiological diagnosis	43 (45.7)	28 (56)	15 (34.1)	.03*
Positive blood culture	14 (14.9)	12 (24)	2 (4.5)	.008*
Potentially drug-resistance bacteria	21 (22.3)	13 (46.4)	8 (53.3)	.36
Combination therapy	57 (60.6)	30 (60)	27 (61.4)	.89
Guideline-concordant antibiotic therapy^a^	54 (57.4)	24 (48)	30 (68.2)	.048*
Guideline- discordant antibiotic therapy:^a^	40 (42.6)	26 (52)	14 (31.8)	
β-lactams without quinolone or macrolide:	33 (35.1)	20 (40)	13 (29.5)	
Nonantipseudomonas β-lactams without quinolone or macrolide	9 (9.6)	4 (8)	5 (11.4)	
Antipseudomonas β-lactams without quinolone or macrolide	24 (25.5)	16 (32)	8 (18.2)	
Quinolone without β-lactams	5 (5.3)	5 (10)	0	
Macrolide without β-lactams	2 (2.1)	1 (2)	1 (2.3)	
Inadequate initial antibiotic treatment^b^	11/43 (25.6)	9/28 (32.1)	2/15 (13.3)	.28
Initial antibiotic regimen was modified	37 (39.4)	26 (52)	11 (25)	.008*
Treatment failure	27 (28.7)	23 (46)	4 (9.1)	<.001*
Death	18 (19.1)	14 (28)	4 (9.1)	.02*
Unfavorable outcome^c^	31 (32.9)	25 (50)	6 (13.6)	<.001*
Hospital-free day, d^d^	15.4 ± 9.8	11.6 ± 9.9	19.7 ± 7.7	<.001*

Data are presented as mean ± SD or n (%), unless otherwise stated. ^a^The 2007 ATS/IDSA guidelines on the management of community-acquired pneumonia in adults. ^b^Pathogens detected were not susceptible to the antibiotics administered within 24 h of presentation. ^c^Unfavorable outcome includes treatment failure or death during hospital admission. ^d^Number of days from admission to day 30 that the patient was not admitted to the hospital. *Statistically significant difference

**Table 5:** Risk factors for Infectious Diseases Society of America/American Thoracic Society Guideline – discordant initial antibiotic selection

	Guidelines Concordance (*n* = 54)	Guidelines Discordance (*n* = 40)	*p*-value
Age, y	68.5 ± 15.9	71.3 ± 19.6	.44
Gender (Female)	28 (51.8)	9 (22.5)	.004*
Severe community-acquired pneumonia	24 (44.4)	26 (65)	.048*
Comorbid conditions:			
None	17 (31.5)	9 (22.5)	.5
Single	22 (40.7)	16 (40)	
Multiple	15 (27.8)	15 (37.5)	
Prior antibiotic therapy^a^	20 (37)	23 (57.5)	.049*
ECOG ≥2^b^	28 (51.8)	26 (65)	.2
Enteral tube feeding	2 (3.7)	7 (17.5)	.03*
Healthcare–associated pneumonia	11 (20.4)	19 (47.5)	.005*
Pleural effusion	11 (20.4)	0	.002*
Initial antibiotic treatment:			
Nonantipseudomonas β-lactams	42 (77.8)	10 (25)	<.001*
Antipseudomonas β-lactams	3 (5.6)	24 (60)	<.001*
Macrolide	43 (79.6)	2 (5)	<.001*
Quinolone	11 (20.4)	5 (12.5)	.31

Data are presented as mean ± SD or n (%), unless otherwise stated

^a^Prior antibiotic therapy: systemic antibiotic use in the 90 days prior to this admission. ^b^ECOG scale: Eastern Cooperative Oncology Group scale of performance status. *Statistically significant difference

### Outcomes

The risk factors for unfavorable outcome in univariate analysis for all 94 patients hospitalized with CAP and 50 patients with severe CAP are shown in Table 6 and Table 7, respectively. Only age and initial antibiotic treatment discordant with IDSA/ATS guidelines for CAP in ICU were found to be independent risk factors for unfavorable outcome in multivariate logistic regression analysis as shown in Table 8. Of note, the initial antibiotic selections prescribed in patients who had unfavorable outcome were modified during the course of treatment more frequently than the initial antibiotic selections in patients who had favorable outcome. In addition, patients with unfavorable outcome had significantly lower number of hospital-free days at 30 days after enrollment into the study than patients with favorable outcome as shown in Table 7.

**Table 6:** Clinical characteristics and clinical outcomes of all patients hospitalized with community-acquired pneumonia (CAP) and patients with severe CAP

	All Patients			Severe CAP		
	Favorable Outcome^†^ (*n* = 63)	Unfavorable Outcome^†^ (*n* = 31)	*p*-value	Favorable Outcome^†^ (*n* = 25)	Unfavorable Outcome^†^ (*n* = 25)	*p*-value
Age, y	65.6 ± 18.5	77.9 ± 11.8	<.001*	65.5 ± 15.7	76.4 ± 11.7	.008*
Gender (Female)	25 (39.7)	12 (38.7)	.93	9 (36)	9 (36)	1
Comorbid conditions:			.04*			.65
None	22 (34.9)	4 (12.9)		6 (24)	3 (12)	
Single	25 (39.7)	13 (41.9)		10 (40)	11 (44)	
Multiple	16 (25.4)	14 (45.2)		9 (36)	11 (44)	
Prior antibiotic therapy^a^	29 (46)	14 (45.2)	.94	10 (40)	11 (44)	.77
ECOG ≥2^b^	31 (49.2)	23 (74.2)	.02*	11 (44)	7 (28)	.24
Enteral tube feeding	5 (7.9)	4 (12.9)	.47	3 (12)	3 (12)	1
HCAP^c^	20 (31.7)	10 (32.3)	.96	11 (44)	7 (28)	.24
Pleural effusion	7 (11.1)	4 (12.9)	1	0	3 (12)	.23
Albumin, g/dL	3 ± 0.5	2.9 ± 0.6	.24	2.9 ± 0.5	2.8 ± 0.7	.84
Globulin, g/dL	3.9 ± 0.6	3.7 ± 0.9	.26	3.7 ± 0.6	3.6 ± 1	.79

Data are presented as mean ± SD or n (%), unless otherwise stated. ^†^Unfavorable outcome includes treatment failure or death during hospital admission. ^a^Prior antibiotic therapy: systemic antibiotic use in the 90 days prior to this admission. ^b^ECOG scale: Eastern Cooperative Oncology Group scale of performance status

^c^HCAP: healthcare–associated pneumonia. *Statistically significant difference

**Table 7:** Initial antibiotics treatment and clinical outcomes of all patients hospitalized with community-acquired pneumonia (CAP) and patients with severe CAP

Initial antibiotics treatment	All Patients			Severe CAP		
	Favorable Outcome^†^ (*n* = 63)	Unfavorable Outcome^†^ (*n* = 31)	*p*-value	Favorable Outcome^†^ (*n* = 25)	Unfavorable Outcome^†^ (*n* = 25)	*p*-value
Antibiotic classes:						
Nonantipseudomonas β-lactams	37 (58.7)	15 (48.4)	.38	14 (56)	12 (48)	.57
Antipseudomonas β-lactams	15 (23.8)	12 (38.7)	.13	8 (32)	10 (40)	.56
Macrolide (M)	35 (55.6)	10 (32.3)	.03*	16 (64)	7 (28)	.01*
Quinolone (Q)	10 (15.9)	6 (19.3)	.67	2 (8)	5 (20)	.42
Combination therapy	39 (61.9)	18 (58.1)	.72	16 (64)	14 (56)	.56
Guideline-concordant treatment^a^	42 (66.7)	12 (38.7)	.01*	16 (64)	8 (32)	.02*
Guideline discordant treatment:^a^	21 (33.3)	19 (61.3)	.01*	9 (36)	17 (68)	.02*
β-lactams without Q or M:	18 (28.6)	15 (48.4)		7 (28)	13 (52)	
Nonantipseudomonas β-lactams without Q or M	5 (7.9)	4 (12.9)		0	4 (16)	
Antipseudomonas β-lactams without Q or M	13 (20.6)	11 (35.5)		7 (28)	9 (36)	
Q without β-lactams	1 (1.6)	4 (12.9)		1 (4)	4 (16)	
M without β-lactams	2 (3.2)	0		1 (4)	0	
Inadequate initial antibiotic treatment^b^	7 (11.1)	4 (12.9)	1	5 (20)	4 (16)	1
Initial antibiotic regimen was modified	13 (20.6)	24 (77.4)	<.001*	7 (28)	19 (76)	.001*
Length of stay in hospital, d^c^	9.9 ± 8.9	20.2 ± 11.1	.001*	13.5 ± 12.9	20.1 ± 12.1	.16
Hospital-free day, d^d^	20.6 ± 5.6	4.8 ± 7.5	<.001*	18 ± 7.1	5.2 ± 8	<.001*

Data are presented as mean ± SD or n (%), unless otherwise stated. ^†^Unfavorable outcome includes treatment failure or death during hospital admission. ^a^The 2007 ATS/IDSA guidelines on the management of community-acquired pneumonia in adults^7^. ^b^Pathogens detected were not susceptible to the antibiotics administered within 24 h of presentation. ^c^Length of stay in hospital in patients who survived to hospital discharge. ^d^Number of days from admission to day 30 that the patient was not admitted to the hospital. *Statistically significant difference

**Table 8:** Risk factors of unfavorable outcome^†^ in patients hospitalized with community-acquired pneumonia (CAP) by univariate and multivariate analyses

	Univariate analysis	Multivariate analysis	
	*p*-value	*p*-value	OR (95%CI)
ALL hospitalized patients with CAP (*n* = 94)			
Age	<.001*	.002*	1.07 (1.03–1.1)
Comorbid conditions	.04*	NS	
ECOG ≥2^a^	.02*	NS	
Severe CAP	<.001*	.001*	7.9 (2.4–26.3)
Guideline-discordant treatment^b^	.01*	.079	2.5 (0.9–7.1)
Hospitalized patients with severe CAP (*n* = 50)			
Age	.008*	.01*	1.1 (1.01–1.1)
Comorbid conditions	.65	NS	
ECOG ≥2^a^	.24	NS	
Guideline-discordant treatment^b^	.02*	.02*	4.6 (1.3–17.1)

OR: odds ratio. CI: confidence interval

^†^Unfavorable outcome includes treatment failure or death during hospital admission

^a^ECOG scale: Eastern Cooperative Oncology Group scale of performance status

^b^The 2007 ATS/IDSA guidelines on the management of community-acquired pneumonia in adults^7^

*Statistically significant difference. NS: not significant difference

## Discussion

Several criteria have been proposed to define severe CAP. We used IDSA/ATS 2007 criteria to define severe CAP, given its good performance for predicting ICU admission and mortality [24, 25]. Moreover, this criteria is based mainly on severity of pneumonia rather than other factors, such as age or comorbidities, and it is simple to use.

The result of this study revealed a substantial number of patients with severe CAP managed in general wards with a high rate of unfavorable outcome (50%). Mortality and treatment failure are two important clinically relevant outcomes [7] that are consistently measured and evaluated among studies in CAP. Only age and initial antibiotic selection were found to be independent risk factors for unfavorable outcome. Initial antibiotic selection concordant with the guidelines for patients with severe CAP in ICU would likely have reduced the rate of unfavorable outcome in about half of these patients. Apart from mortality, patients with unfavorable outcome required longer hospital stay and more frequent modification of initial antibiotic treatment. These factors also contribute appreciably to a higher cost of treatment.

Comorbid conditions and functional status are important prognostic markers in CAP. However, comorbid conditions and functional status were associated with the unfavorable outcome only in all patients hospitalized with CAP but were not associated with the unfavorable outcome in the subgroup of patients with severe pneumonia. This is most likely explained by the smaller number of patients in the severe pneumonia group. In addition, most of the patients in the severe pneumonia group (82%) had some comorbid conditions and 64% of the patients had low functional status (ECOG ≥2). The limited sample size would make it difficult to assess the impact of comorbid conditions and functional status on unfavorable outcome in the severe pneumonia group.

The processes of care and adherence to initial antibiotic selection guidelines have been associated with improved survival, reduced treatment failure, and reduced duration of mechanical ventilation of patients with severe CAP in the ICU [11–14, 21, 26]. The processes of care in general wards is limited and usually much lower-intensity than the care provided in the ICU. As such, antibiotic selection is the only intervention that can be improved to effectuate parity between general wards and the ICU. This study is the first to focus specifically on the impact of initial antibiotic selection on the outcomes of patients with severe CAP in general wards.

Most international guidelines recommend empirical therapy that is based on the location of care, with specific recommendations to those managed as outpatients, as inpatients, and in ICUs [7–10]. Severity scores were not originally designed to guide antibiotic prescription. Severe CAP was one of the risk factors for guideline-discordant initial antibiotic selection in this study as shown in Table 5. This may be due to unrecognition of severe CAP in general medical wards or lack of knowledge of the guidelines for management of severe CAP. This guideline-discordant antibiotic selection led to a higher rate of unfavorable outcome among severe CAP patients treated in general wards. Apart from severe CAP, other risk factors associated with guideline-discordant initial antibiotic selection were prior antibiotic therapy, enteral tube feeding, HCAP, and treatment with antipseudomonas β-lactams. These factors were related to physician concern about the possible presence of multidrug-resistant pathogens that cause CAP, which is a growing problem in many countries - including our country. Put another way, these physicians actively conclude that their patients are at-risk for specific types of pathogens, and that, therefore, the guidelines may not apply. This undue attention to multidrug-resistant pathogens resulted in their decision to drop the second antibiotic, especially the drug required for coverage against atypical copathogens.

The rate of microbial etiology identification in this study (45.8%) was comparable to that of other studies [7, 27, 28]. The pattern of causative organisms identified in this study was more similar to hospital-acquired pneumonia than community-acquired infection, with high prevalence of potentially drug-resistant bacteria. There are many reports, especially from Asian countries, of increasing prevalence of potentially drug-resistant bacteria in CAP [29]. However, most of these potentially drug-resistant bacteria were identified by sputum culture. As such, these culture findings may partly represent colonization. Because this study was a single center study in general medical wards with substantial number of critically illed patients, with small sample size, it may not represent the overall epidemiologic data of the etiology of CAP in general medical wards.

It is not clear how the initial antibiotic regimens according to IDSA/ATS guidelines achieved its beneficial effect for patients with severe CAP in this study. Guideline compliance may only be a surrogate marker for quality of care overall. The most common guideline-discordant regimen was β-lactams without quinolone or macrolide, which resulted in lack of coverage for atypical pathogens. We did not investigate for atypical pathogens in this study. However, a previous study from Thailand revealed atypical pathogens to be responsible for about 30% of the patients hospitalized with CAP [30]. Several observational studies suggested that combination therapy improves survival in the subset of the most severe patients with bacteremic pneumococcal infection [31–33]. The patients with severe CAP in this study also had a higher rate of positive blood culture than patients with non-severe CAP. However, only the combination therapy according to the guidelines that included both β-lactams with quinolone or macrolide, was associated with favorable outcome. Most of the patients in this study that received combination therapy according to the guidelines were given macrolides. Macrolides exert broad-ranging immunomodulatory effects, and its combination with other antibiotics may explain the higher rates of favorable outcome [34].

The present study has several potential limitations. First, our study was a secondary analysis of an observational study with limited sample size, and the results may be subject to the effects of confounders that were not controlled for in regression analyses. Second, only one process (initial antibiotic selection) was analyzed – not composite processes. It was, therefore, not possible to determine whether or not guideline-concordant antibiotic selection was associated with better overall care, or how this affected the results of this study. Third, therapeutic changes introduced after the initial antibiotic treatment was prescribed were not investigated; however, a study reported that such changes may not influence the final outcome [35]. Fourth, the time from admission to first administration of antibiotics has been suggested as a key predictor of outcome, but this information was not evaluated in the present study.

## Conclusions

A substantial number of patients with severe CAP were managed in general wards with a high rate of unfavorable outcome. Adherence to the IDSA/ATS guidelines for initial antibiotic treatment of severe CAP in the ICU may contribute to a lower rate of unfavorable outcome, and a higher number of hospital-free days. Severe CAP outside ICU should be recognized for appropriate initial antibiotic selection to improve outcomes.

## Data Availability

The datasets used in this study are available from the corresponding author upon reasonable request.

## Abbreviations

ATS: American Thoracic Society
CAP: community-acquired pneumonia
ECOG: Eastern Cooperative Oncology Group
HCAP: healthcare-associated pneumonia
IDSA: Infectious Diseases Society of America
